# Supplementary figures and images for: Malignant Melanoma: An Overview, New Perspectives, and Vitamin D Signaling
Source: Cancers (Basel). 2024 Jun 18;16(12):2262. doi: 10.3390/cancers16122262 (PMC11201527; doi:10.3390/cancers16122262)

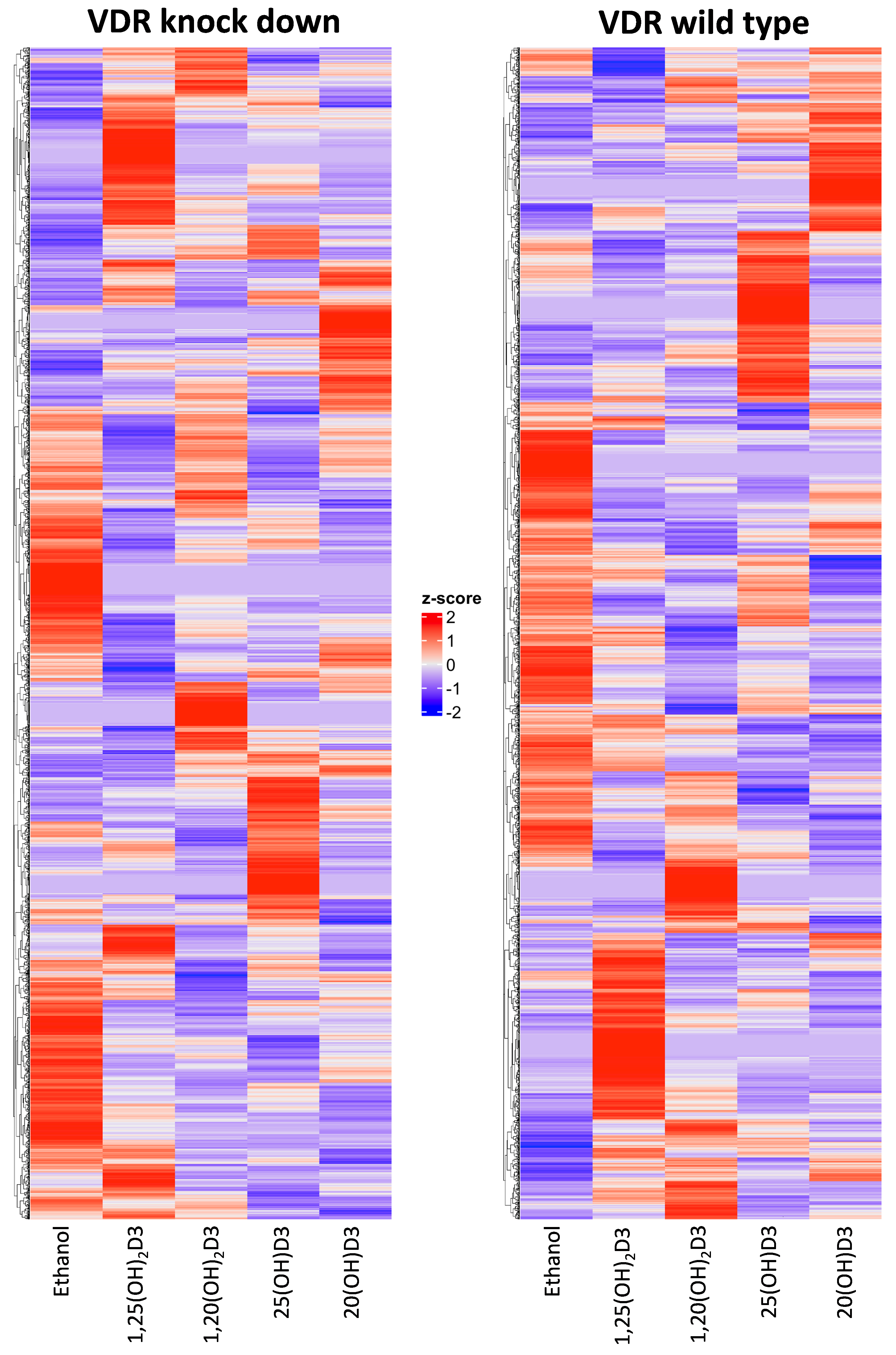

Supplement: Supplementary file 1 [file cancers-16-02262-s001.zip › Supplemental figure 1.tif]

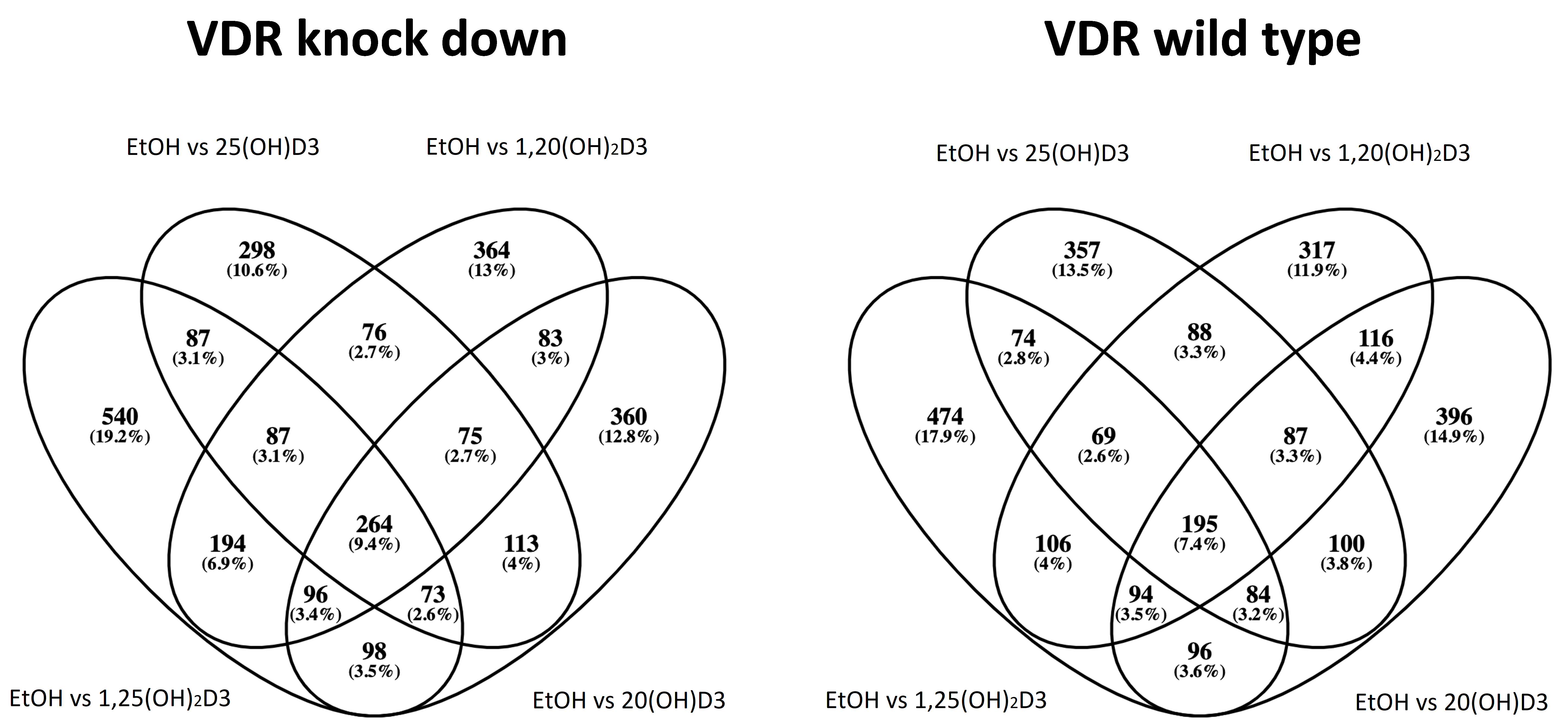

Supplement: Supplementary file 1 [file cancers-16-02262-s001.zip › Supplemental figure 2.tif]
